# (*Z*)-2-(2-Hy­droxy-4-meth­oxy­benzyl­idene)-1-benzofuran-3(2*H*)-one

**DOI:** 10.1107/S1600536811016217

**Published:** 2011-05-07

**Authors:** J. Satyanarayana Reddy, N. Ravi Kumar, J. Venkata Prasad, G. Gopikrishna, K. Anand Solomon

**Affiliations:** a Sankar Foundation Research Institute, Naiduthota, Vepagunta, Visakhapatnam, Andhra Pradesh 530 047, India

## Abstract

In the title compound, C_16_H_12_O_4_, the 1-benzofuran­one unit is in a planar conformation [C—C—C—C = 179.69 (12)°]. The conformation around the C=C double bond [1.3370 (17) Å] is *Z*. In the crystal, the mol­ecules are stabilized by O—H⋯O (running parallel to the *bc* plane) and C—H⋯O hydrogen bonds.

## Related literature

For the synthesis and biological activity of substituted aurones, see: Varma & Varma (1992 ▶); Beney *et al.* (2001 ▶); Sim *et al.* (2008 ▶); Souard *et al.* (2010 ▶); Wang *et al.* (2007 ▶). For aurones as structural scaffolds in natural and synthetic compounds possessing diverse biological properties, see: Villemin *et al.* (1998 ▶). The title compound, which is an analogue of naturally occurring aurones, holds promise as an inhibitor against human melanocytes tyrosinase towards anti­hyper­pig­ment­ation, see: Okombi *et al.* (2006 ▶). For the assignment of conformations and the orientation of the substituents, see: Nardelli (1983 ▶, 1995 ▶); Klyne & Prelog (1960 ▶).
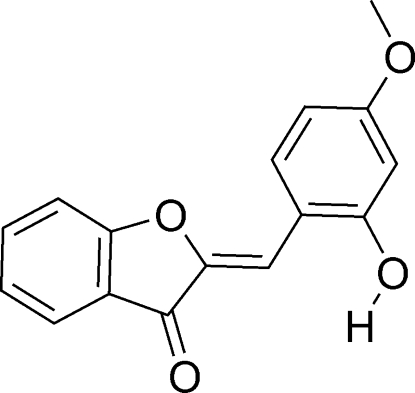


## Experimental

### 

#### Crystal data

C_16_H_12_O_4_
*M* *_r_* = 268.26
Monoclinic, 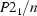

*a* = 7.1083 (4) Å
*b* = 12.7072 (7) Å
*c* = 14.4024 (8) Å
β = 100.161 (2)°
*V* = 1280.52 (12) Å^3^
*Z* = 4
Mo *K*α radiation
μ = 0.10 mm^−1^
*T* = 293 K
0.35 × 0.30 × 0.25 mm

#### Data collection

Bruker Kappa APEXII CCD diffractometer
Absorption correction: multi-scan (*SADABS*; Bruker, 2004 ▶) *T*_min_ = 0.906, *T*_max_ = 0.975
19357 measured reflections
4765 independent reflections
2533 reflections with *I* > 2σ(*I*)
*R*_int_ = 0.047

#### Refinement

*R*[*F* ^2^ > 2σ(*F* ^2^)] = 0.052
*wR*(*F* ^2^) = 0.150
*S* = 1.02
4765 reflections
187 parameters
H atoms treated by a mixture of independent and constrained refinement
Δρ_max_ = 0.26 e Å^−3^
Δρ_min_ = −0.19 e Å^−3^

### 

Data collection: *APEX2* (Bruker, 2004 ▶); cell refinement: *SAINT* (Bruker, 2004 ▶); data reduction: *SAINT* and *XPREP* (Bruker, 2004 ▶); program(s) used to solve structure: *SIR92* (Altomare *et al.*, 1993 ▶); program(s) used to refine structure: *SHELXL97* (Sheldrick, 2008 ▶); molecular graphics: *ORTEP-3* (Farrugia, 1997 ▶) and *Mercury* (Macrae *et al.*, 2006 ▶); software used to prepare material for publication: *PLATON* (Spek, 2009 ▶).

## Supplementary Material

Crystal structure: contains datablocks I, global. DOI: 10.1107/S1600536811016217/ds2109sup1.cif

Structure factors: contains datablocks I. DOI: 10.1107/S1600536811016217/ds2109Isup2.hkl

Supplementary material file. DOI: 10.1107/S1600536811016217/ds2109Isup3.cml

Additional supplementary materials:  crystallographic information; 3D view; checkCIF report

## Figures and Tables

**Table 1:** Hydrogen-bond geometry (Å, °)

*D*—H⋯*A*	*D*—H	H⋯*A*	*D*⋯*A*	*D*—H⋯*A*
O3—H3*A*⋯O2^i^	0.90 (2)	1.80 (2)	2.6952 (14)	170.0 (19)
C16—H16*A*⋯O3^i^	0.96	2.59	3.3328 (14)	135

Symmetry code: (i) 
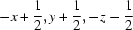
.

**Fig. 1.**
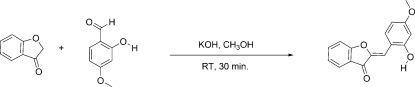
Reaction scheme.

**Fig. 2.**
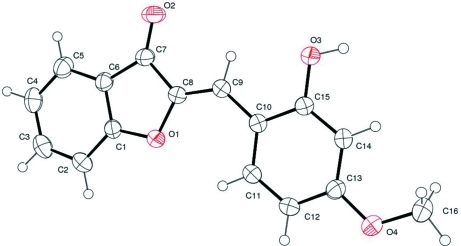
ORTEP diagram of (Z)-2-(2-hydroxy-4-methoxybenzylidene)benzofuran-3(2H)-one. (Thermal ellipsoids are at 50% probability level).

**Fig. 3.**
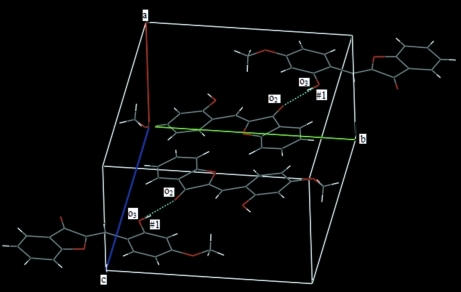
Crystal packing diagram of the title compound. Symmetry codes -x + 1/2,y + 1/2,-z - 1/2

## supplementary crystallographic information

### Comment

Aurones are chalcone analogues containing fused benzofuranone ring system. They form essential structural scaffold in several natural and synthetic molecules possessing diverse biological properties (Villemin *et al.* 1998) Several functionalized aurones were reported to exhibit anti-malarial (Souard *et al.* 2010) and anti-histamine (Wang *et al.* 2007) properties. The title compound which is an analogue of naturally occurring aurones holds promise as inhibitors against human melanocytes tyrosinase towards antihyperpigmentation (Okombi *et al.* 2006).

### Experimental

3-coumaranone was allowed to react with 2-hydroxy-4-methoxybenzaldehyde (aldol condensation) in alcoholic solution in the presence of potassium hydroxide for 30 minutes to yield the title compound. The pure product was obtained by recrystalizing the crude product in ethanol solvent.

### Refinement

Refinement of F^2^ against ALL reflections. The weighted *R*-factor *wR* and goodness of fit S are based on F^2^, conventional *R*-factors *R* are based on F, with F set to zero for negative F^2^. The threshold expression of F^2^ > 2sigma(F^2^) is used only for calculating *R*-factors(gt) *etc*. and is not relevant to the choice of reflections for refinement. *R*-factors based on F^2^ are statistically about twice as large as those based on F, and R– factors based on ALL data will be even larger.

### Crystal data

**Table:**

C_16_H_12_O_4_	*F*(000) = 560
*M**_r_* = 268.26	*D*_x_ = 1.391 Mg m^−^^3^
Monoclinic, *P*2_1_/*n*	Mo *K*α radiation, λ = 0.71073 Å
Hall symbol: -P 2yn	Cell parameters from 3249 reflections
*a* = 7.1083 (4) Å	θ = 2.9–25.3°
*b* = 12.7072 (7) Å	µ = 0.10 mm^−^^1^
*c* = 14.4024 (8) Å	*T* = 293 K
β = 100.161 (2)°	Block, yellow
*V* = 1280.52 (12) Å^3^	0.35 × 0.30 × 0.25 mm
*Z* = 4	

### Data collection

**Table:**

Bruker Kappa APEXII CCD diffractometer	4765 independent reflections
Radiation source: fine-focus sealed tube	2533 reflections with *I* > 2σ(*I*)
graphite	*R*_int_ = 0.047
ω and φ scans	θ_max_ = 32.9°, θ_min_ = 2.2°
Absorption correction: multi-scan (*SADABS*; Bruker, 2004)	*h* = −10→5
*T*_min_ = 0.906, *T*_max_ = 0.975	*k* = −17→19
19357 measured reflections	*l* = −21→21

### Refinement

**Table:**

Refinement on *F*^2^	Secondary atom site location: difference Fourier map
Least-squares matrix: full	Hydrogen site location: inferred from neighbouring sites
*R*[*F*^2^ > 2σ(*F*^2^)] = 0.052	H atoms treated by a mixture of independent and constrained refinement
*wR*(*F*^2^) = 0.150	*w* = 1/[σ^2^(*F*_o_^2^) + (0.0683*P*)^2^ + 0.0121*P*] where *P* = (*F*_o_^2^ + 2*F*_c_^2^)/3
*S* = 1.02	(Δ/σ)_max_ = 0.001
4765 reflections	Δρ_max_ = 0.26 e Å^−^^3^
187 parameters	Δρ_min_ = −0.19 e Å^−^^3^
0 restraints	Extinction correction: *SHELXL97* (Sheldrick, 2008), Fc^*^=kFc[1+0.001xFc^2^λ^3^/sin(2θ)]^-1/4^
Primary atom site location: structure-invariant direct methods	Extinction coefficient: 0.0043 (16)

### Special details

**Table:**

Geometry. All e.s.d.'s (except the e.s.d. in the dihedral angle between two l.s. planes) are estimated using the full covariance matrix. The cell e.s.d.'s are taken into account individually in the estimation of e.s.d.'s in distances, angles and torsion angles; correlations between e.s.d.'s in cell parameters are only used when they are defined by crystal symmetry. An approximate (isotropic) treatment of cell e.s.d.'s is used for estimating e.s.d.'s involving l.s. planes.
Refinement. Refinement of *F*^2^ against ALL reflections. The weighted *R*-factor *wR* and goodness of fit *S* are based on *F*^2^, conventional *R*-factors *R* are based on *F*, with *F* set to zero for negative *F*^2^. The threshold expression of *F*^2^ > σ(*F*^2^) is used only for calculating *R*-factors(gt) *etc*. and is not relevant to the choice of reflections for refinement. *R*-factors based on *F*^2^ are statistically about twice as large as those based on *F*, and *R*- factors based on ALL data will be even larger.

### Fractional atomic coordinates and isotropic or equivalent isotropic displacement parameters (Å^2^)

**Table:**

	*x*	*y*	*z*	*U*_iso_*/*U*_eq_	
C1	0.33155 (18)	0.81587 (10)	0.10935 (9)	0.0378 (3)	
C2	0.3577 (2)	0.79394 (12)	0.20448 (10)	0.0498 (4)	
H2	0.3493	0.8460	0.2490	0.060*	
C3	0.3967 (2)	0.69092 (12)	0.22996 (11)	0.0544 (4)	
H3	0.4168	0.6732	0.2936	0.065*	
C4	0.4072 (2)	0.61232 (12)	0.16407 (11)	0.0529 (4)	
H4	0.4321	0.5433	0.1840	0.064*	
C5	0.3811 (2)	0.63594 (11)	0.06989 (11)	0.0487 (4)	
H5	0.3888	0.5838	0.0254	0.058*	
C6	0.34267 (17)	0.73990 (10)	0.04224 (9)	0.0383 (3)	
C7	0.31090 (19)	0.79320 (10)	−0.04815 (9)	0.0409 (3)	
C8	0.27856 (18)	0.90379 (10)	−0.02558 (9)	0.0380 (3)	
C9	0.24089 (17)	0.98235 (10)	−0.08784 (9)	0.0388 (3)	
H9	0.2352	0.9621	−0.1503	0.047*	
C10	0.20767 (16)	1.09256 (10)	−0.07510 (9)	0.0362 (3)	
C11	0.2172 (2)	1.14095 (10)	0.01304 (10)	0.0440 (3)	
H11	0.2418	1.0999	0.0673	0.053*	
C12	0.1913 (2)	1.24709 (11)	0.02151 (10)	0.0492 (4)	
H12	0.1987	1.2772	0.0809	0.059*	
C13	0.15384 (19)	1.30956 (10)	−0.05866 (10)	0.0412 (3)	
C14	0.14158 (17)	1.26536 (10)	−0.14673 (9)	0.0389 (3)	
H14	0.1155	1.3072	−0.2004	0.047*	
C15	0.16833 (18)	1.15787 (10)	−0.15485 (9)	0.0383 (3)	
C16	0.0907 (2)	1.48154 (11)	−0.12239 (11)	0.0557 (4)	
H16A	0.1933	1.4791	−0.1577	0.083*	
H16B	0.0756	1.5523	−0.1015	0.083*	
H16C	−0.0256	1.4589	−0.1617	0.083*	
O1	0.29265 (13)	0.91457 (7)	0.07146 (6)	0.0425 (2)	
O2	0.30983 (16)	0.75813 (8)	−0.12769 (7)	0.0611 (3)	
O3	0.15733 (16)	1.11203 (8)	−0.24045 (7)	0.0558 (3)	
O4	0.13342 (16)	1.41402 (8)	−0.04288 (7)	0.0556 (3)	
H3A	0.153 (3)	1.1617 (15)	−0.2855 (16)	0.092 (7)*	

### Atomic displacement parameters (Å^2^)

**Table:**

	*U*^11^	*U*^22^	*U*^33^	*U*^12^	*U*^13^	*U*^23^
C1	0.0425 (6)	0.0355 (7)	0.0351 (7)	−0.0041 (5)	0.0061 (5)	0.0037 (6)
C2	0.0625 (9)	0.0539 (9)	0.0334 (7)	−0.0009 (7)	0.0092 (6)	0.0034 (7)
C3	0.0590 (9)	0.0622 (10)	0.0417 (8)	0.0000 (7)	0.0081 (7)	0.0153 (8)
C4	0.0516 (8)	0.0457 (9)	0.0598 (10)	0.0006 (6)	0.0052 (7)	0.0184 (7)
C5	0.0538 (8)	0.0382 (8)	0.0518 (9)	0.0018 (6)	0.0032 (6)	0.0015 (7)
C6	0.0413 (6)	0.0349 (7)	0.0370 (7)	−0.0023 (5)	0.0021 (5)	0.0023 (5)
C7	0.0511 (7)	0.0367 (7)	0.0333 (7)	−0.0009 (6)	0.0029 (5)	−0.0028 (6)
C8	0.0476 (7)	0.0364 (7)	0.0299 (6)	−0.0026 (5)	0.0065 (5)	−0.0015 (5)
C9	0.0496 (7)	0.0357 (7)	0.0314 (6)	−0.0021 (5)	0.0079 (5)	−0.0001 (5)
C10	0.0419 (6)	0.0339 (7)	0.0339 (7)	−0.0024 (5)	0.0099 (5)	0.0007 (5)
C11	0.0608 (8)	0.0390 (7)	0.0347 (7)	0.0017 (6)	0.0154 (6)	0.0027 (6)
C12	0.0736 (9)	0.0420 (8)	0.0361 (8)	0.0030 (7)	0.0212 (7)	−0.0029 (6)
C13	0.0490 (7)	0.0324 (7)	0.0455 (8)	0.0011 (5)	0.0175 (6)	−0.0015 (6)
C14	0.0476 (7)	0.0336 (7)	0.0362 (7)	−0.0005 (5)	0.0096 (5)	0.0043 (5)
C15	0.0463 (7)	0.0354 (7)	0.0337 (7)	−0.0051 (5)	0.0086 (5)	−0.0018 (5)
C16	0.0749 (10)	0.0352 (8)	0.0597 (10)	0.0034 (7)	0.0194 (8)	0.0045 (7)
O1	0.0612 (6)	0.0353 (5)	0.0313 (5)	−0.0006 (4)	0.0092 (4)	0.0004 (4)
O2	0.1001 (9)	0.0452 (6)	0.0353 (6)	0.0094 (5)	0.0048 (5)	−0.0087 (5)
O3	0.1003 (8)	0.0346 (6)	0.0317 (5)	−0.0049 (5)	0.0092 (5)	−0.0017 (4)
O4	0.0859 (7)	0.0353 (6)	0.0495 (6)	0.0077 (5)	0.0222 (5)	−0.0011 (5)

### Geometric parameters (Å, °)

**Table:**

C1—O1	1.3764 (15)	C9—H9	0.9300
C1—C6	1.3781 (19)	C10—C11	1.4014 (18)
C1—C2	1.3782 (19)	C10—C15	1.4046 (18)
C2—C3	1.374 (2)	C11—C12	1.3696 (18)
C2—H2	0.9300	C11—H11	0.9300
C3—C4	1.389 (2)	C12—C13	1.3876 (19)
C3—H3	0.9300	C12—H12	0.9300
C4—C5	1.370 (2)	C13—O4	1.3588 (16)
C4—H4	0.9300	C13—C14	1.3758 (18)
C5—C6	1.3927 (18)	C14—C15	1.3868 (18)
C5—H5	0.9300	C14—H14	0.9300
C6—C7	1.4495 (18)	C15—O3	1.3533 (16)
C7—O2	1.2280 (16)	C16—O4	1.4203 (17)
C7—C8	1.4697 (18)	C16—H16A	0.9600
C8—C9	1.3370 (17)	C16—H16B	0.9600
C8—O1	1.3902 (15)	C16—H16C	0.9600
C9—C10	1.4373 (17)	O3—H3A	0.90 (2)
			
O1—C1—C6	113.12 (11)	C11—C10—C15	116.89 (12)
O1—C1—C2	124.11 (12)	C11—C10—C9	124.08 (12)
C6—C1—C2	122.77 (12)	C15—C10—C9	119.01 (11)
C3—C2—C1	116.33 (14)	C12—C11—C10	121.80 (13)
C3—C2—H2	121.8	C12—C11—H11	119.1
C1—C2—H2	121.8	C10—C11—H11	119.1
C2—C3—C4	122.35 (14)	C11—C12—C13	119.89 (13)
C2—C3—H3	118.8	C11—C12—H12	120.1
C4—C3—H3	118.8	C13—C12—H12	120.1
C5—C4—C3	120.32 (14)	O4—C13—C14	124.18 (12)
C5—C4—H4	119.8	O4—C13—C12	115.47 (12)
C3—C4—H4	119.8	C14—C13—C12	120.34 (12)
C4—C5—C6	118.46 (14)	C13—C14—C15	119.51 (12)
C4—C5—H5	120.8	C13—C14—H14	120.2
C6—C5—H5	120.8	C15—C14—H14	120.2
C1—C6—C5	119.77 (13)	O3—C15—C14	120.93 (12)
C1—C6—C7	106.47 (11)	O3—C15—C10	117.50 (12)
C5—C6—C7	133.75 (13)	C14—C15—C10	121.57 (12)
O2—C7—C6	130.00 (13)	O4—C16—H16A	109.5
O2—C7—C8	125.30 (13)	O4—C16—H16B	109.5
C6—C7—C8	104.70 (11)	H16A—C16—H16B	109.5
C9—C8—O1	124.83 (12)	O4—C16—H16C	109.5
C9—C8—C7	125.89 (12)	H16A—C16—H16C	109.5
O1—C8—C7	109.29 (10)	H16B—C16—H16C	109.5
C8—C9—C10	131.29 (12)	C1—O1—C8	106.42 (10)
C8—C9—H9	114.4	C15—O3—H3A	110.1 (13)
C10—C9—H9	114.4	C13—O4—C16	117.98 (11)
			
O1—C1—C2—C3	−179.59 (12)	C8—C9—C10—C11	2.7 (2)
C6—C1—C2—C3	0.2 (2)	C8—C9—C10—C15	−179.18 (13)
C1—C2—C3—C4	−0.8 (2)	C15—C10—C11—C12	−0.59 (19)
C2—C3—C4—C5	0.9 (2)	C9—C10—C11—C12	177.54 (13)
C3—C4—C5—C6	−0.4 (2)	C10—C11—C12—C13	0.2 (2)
O1—C1—C6—C5	−179.94 (11)	C11—C12—C13—O4	−178.96 (13)
C2—C1—C6—C5	0.23 (19)	C11—C12—C13—C14	0.4 (2)
O1—C1—C6—C7	0.83 (14)	O4—C13—C14—C15	178.78 (12)
C2—C1—C6—C7	−178.99 (12)	C12—C13—C14—C15	−0.47 (19)
C4—C5—C6—C1	−0.12 (19)	C13—C14—C15—O3	−179.95 (11)
C4—C5—C6—C7	178.85 (14)	C13—C14—C15—C10	0.05 (18)
C1—C6—C7—O2	179.03 (14)	C11—C10—C15—O3	−179.53 (11)
C5—C6—C7—O2	0.0 (3)	C9—C10—C15—O3	2.24 (17)
C1—C6—C7—C8	−0.86 (14)	C11—C10—C15—C14	0.47 (18)
C5—C6—C7—C8	−179.93 (14)	C9—C10—C15—C14	−177.76 (11)
O2—C7—C8—C9	0.9 (2)	C6—C1—O1—C8	−0.43 (14)
C6—C7—C8—C9	−179.20 (12)	C2—C1—O1—C8	179.39 (12)
O2—C7—C8—O1	−179.27 (13)	C9—C8—O1—C1	179.68 (12)
C6—C7—C8—O1	0.63 (14)	C7—C8—O1—C1	−0.15 (13)
O1—C8—C9—C10	0.5 (2)	C14—C13—O4—C16	2.1 (2)
C7—C8—C9—C10	−179.69 (12)	C12—C13—O4—C16	−178.64 (13)

### Hydrogen-bond geometry (Å, °)

**Table:**

*D*—H···*A*	*D*—H	H···*A*	*D*···*A*	*D*—H···*A*
O3—H3A···O2^i^	0.90 (2)	1.80 (2)	2.6952 (14)	170.0 (19)
C16—H16A···O3^i^	0.96	2.59	3.3328 (14)	135

Symmetry codes: (i) −*x*+1/2, *y*+1/2, −*z*−1/2.
